# Assessing leaf nitrogen concentration in rice using RGB imaging: a comparative study at leaf, canopy, and plot scales

**DOI:** 10.3389/fpls.2025.1599177

**Published:** 2025-08-05

**Authors:** Haixiao Ge, Gaoqiang Lv, Yang Qin, Min Shen

**Affiliations:** ^1^ College of Rural Revitalization, Jiangsu Open University, Nanjing, China; ^2^ College of Agricultural Engineering, Jiangsu University, Zhenjiang, China

**Keywords:** LNC, RGB imaging, rice, SMLR, plot scale

## Abstract

Leaf nitrogen concentration (LNC) is a critical indicator for evaluating crop health and optimizing nitrogen management in sustainable agriculture. While multispectral and hyperspectral sensing techniques enable precise LNC estimation, their high cost and technical complexity often hinder practical application. This study assesses RGB imaging as a cost-effective and accessible alternative for estimating rice LNC across leaf, canopy, and plot scales. Field experiments conducted at two sites during the 2018–2019 reproductive stages acquired RGB images at three spatial resolutions. For canopy and plot images, rice vegetation was isolated using green minus red (GMR) band indices and thresholding. Stepwise multiple linear regression (SMLR) models incorporating 13 color indices were developed. Results demonstrated that leaf-scale models achieved superior accuracy (R^2^ = 0.84-0.87, RMSE = 0.16-0.25%), validating RGB imaging’s potential for high-precision diagnostics. At the canopy scale, vegetation segmentation enhanced model performance (an average R^2^ increase of 3% compared to those from unsegmented images), confirming the necessity of background removal. Plot-scale analysis revealed that UAV flight altitude minimally affected model accuracy within the range tested, with 100 m yielding comparable performance (R^2^ = 0.61-0.65) to other altitudes. Cross-site validation indicated promising generalizability at the leaf scale, while canopy and plot scale models exhibited greater sensitivity to environmental variations. This research establishes RGB imaging as a scalable tool for rice nitrogen monitoring, demonstrating that segmentation improves accuracy at larger spatial scales. These findings provide practical insights for implementing precision nitrogen management in smallholder farming systems, supporting ecological sustainability through reduced fertilizer overuse.

## 1 Introduction

Leaf nitrogen concentration (LNC, %) is a critical biophysical parameter for diagnosing crop nitrogen status and guiding precision fertilization. As a direct proxy of photosynthetic capacity and metabolic activity, LNC reflects real-time plant nitrogen assimilation efficiency, making it indispensable for optimizing fertilizer use efficiency (Lu et al., 2021; Zhang et al., 2024). Traditional methods for LNC quantification rely on destructive sampling followed by laboratory Kjeldahl analysis. This process is not only labor-intensive but also introduces significant time lags between sampling and results (Padilla et al., 2014). Such delays hinder timely field interventions, particularly during rapid growth stages like booting or flowering.

Remote sensing technologies have emerged as transformative alternatives by enabling non-destructive, real-time LNC estimation through spectral reflectance measurements. Platforms spanning satellites, unmanned aerial vehicles (UAVs), and ground-based sensors now provide multi-scale monitoring capabilities. For instance, satellite-derived vegetation indices (VIs) such as the normalized difference vegetation index (NDVI) and the enhanced vegetation index (EVI) facilitate regional-scale LNC assessment (Kalacska et al., 2015; Lepine et al., 2016), while Sentinel-2’s red-edge bands enhance nitrogen-sensitive spectral retrievals (Crema et al., 2020). However, satellite-based approaches face inherent limitations: coarse spatial resolutions obscure field-level heterogeneity, cloud cover frequently compromises data availability, and infrequent revisit cycles miss critical phenological transitions (Li et al., 2010). These constraints necessitate complementary proximal sensing solutions.

Proximal remote sensing platforms, including UAV-mounted multispectral or hyperspectral sensors and tractor-integrated active canopy sensors (e.g., GreenSeeker, Crop Circle), address satellite limitations by delivering high spatiotemporal resolution (sub-meter to centimeter scale) and cloud-independent operation (Dai et al., 2023; Xu et al., 2023). These systems leverage red and near-infrared (NIR) spectral regions to calculate VIs like the normalized difference red edge index (NDRE) and the chlorophyll index red edge (CIRE), which strongly correlate with LNC in crops such as rice and wheat (Erdle et al., 2011; Li et al., 2012). Advanced machine learning techniques, including random forests and neural networks, further enhance prediction accuracy by integrating multi-sensor data (Feng et al., 2014; He et al., 2016). Despite their technical advantages, these systems face two critical barriers to widespread adoption. First, the high costs of multispectral/hyperspectral sensors and UAV platforms render them economically unviable for smallholder farmers, who dominate agricultural production in regions like southern China (Nirere et al., 2022; Shi et al., 2021; Tian et al., 2022). Second, their operation demands specialized expertise in sensor calibration, data processing, and spectral analysis—skills often lacking in resource-limited settings. These challenges underscore the urgent need for affordable, user-friendly alternatives that democratize precision nitrogen management.

RGB imaging has re-emerged as a promising low-cost solution, building upon the legacy of the Leaf Color Chart (LCC)—a simple, color-based tool widely adopted since the 1990s for visual nitrogen status diagnosis (Shukla et al., 2004). Modern RGB systems leverage consumer-grade cameras and computational algorithms to extract quantitative color indices from red, green, and blue channel digital numbers (DNs). For example, the normalized redness intensity (NRI) and excess green index (ExG) have demonstrated strong correlations with LNC in maize and rice (Lu et al., 2021; Zhang et al., 2020). Recent studies further integrate machine learning techniques with multiple VIs to improve model robustness across varying lighting conditions and growth stages (Shi et al., 2021). However, existing research predominantly focuses on single spatial scales—either leaf-level (using controlled lighting scanners) or canopy-level (using handheld cameras)—overlooking the hierarchical nature of agricultural monitoring. This oversight is critical because LNC variability manifests differently across scales: leaf-level measurements capture physiological status, canopy-level integrates plant architecture effects, and plot-level incorporates field heterogeneity (Xu et al., 2024). Ignoring these scale-dependent dynamics risks oversimplifying model interpretations and limiting operational utility. Moreover, canopy- and plot-scale RGB imaging faces unique challenges, such as background interference from soil and water, which are rarely addressed systematically.

The specific objectives of this research are threefold: (1) to develop stepwise multiple linear regression (SMLR) models by integrating 13 color indices from three RGB devices (flatbed scanner, digital camera, and UAV), and evaluate their predictive power across different scales; (2) to quantify performance variations (in terms of R^2^ and RMSE) among leaf-, canopy-, and plot-scale models via cross-validation, and identify optimal spatial resolutions for diverse end-users; (3) to assess the efficacy of the green-minus-red (GMR) threshold segmentation technique in mitigating background noise at canopy and plot scales, and evaluate its impact on model accuracy and generalizability.

By clarifying scale-dependent correlations between RGB-derived indices and LNC, this study advances scalable precision nitrogen management tools. The findings directly inform the design of cost-effective monitoring systems for smallholder farming, balancing ecological sustainability with yield security.

## 2 Materials and methods

### 2.1 Experiment design

**Figure 1:**
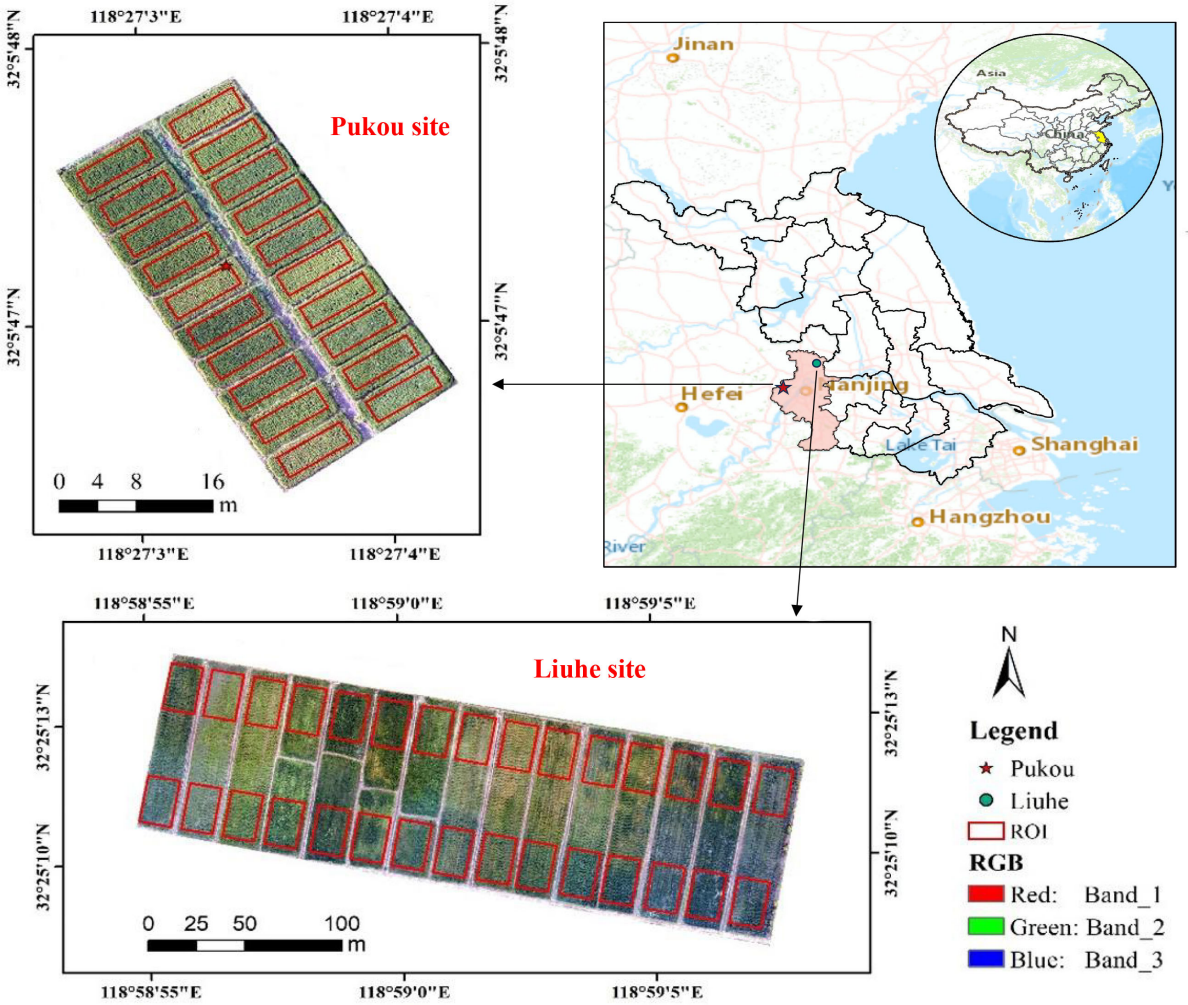
Geographical distribution of the experimental sites. **(A)** Jiangsu Province, **(B)** Pukou site, **(C)** Liuhe site.

The two field experiments for this study were conducted in the Pukou District (32°04’15” N, 118°28’21” E) and Liuhe District (32°25’04” N, 118°59’18” E) of Nanjing City, Jiangsu Province, China (**Figure 1**). Each experiment was organized into treatment plots with multiple replications to study the effects of varying rates and types of nitrogen input (**Table 1**). Specifically, the Pukou experiment consisted of five treatments that examined different rates of controlled-release fertilizer input, each with four replications (20 plots in total), while the Liuhe experiment involved four treatments, each with four replications except for the control (CK), which had three replications, resulting in a total of 15 plots (each measuring 200 m²).

**Table 1:** Fertilization information of field experiments in this study.

Experimental site	Fertilization treatment	Compound fertilizer (kg N ha^-1^)	Proportion of controlled release N
Pukou	N1	0	0%
	N2	240	0%
	N3	240	30%
	N4	240	40%
	N5	240	50%
Liuhe	N1	158	50%
	N2	176	50%
	N3	196	50%
	N4	196	0%

The experimental sites in Pukou and Liuhe featured paddy soils (anthrosols) with distinct fertility profiles. Pukou soils exhibited organic matter content of 22.3 g kg^-1^, total nitrogen of 1.3 g kg^-1^, Olsen phosphorus of 15.4 mg kg^-1^, and available potassium of 146.4 mg kg^-1^. Liuhe soils contained higher nutrient levels: 26.6 g kg^-1^ organic matter, 1.6 g kg^-1^ total nitrogen, 15.2 mg kg^-1^ Olsen phosphorus, and 166.2 mg kg^-1^ available potassium. Regarding agronomic practices, two japonica rice cultivars (Wuyunjing 23 and Nanjing 5055) were transplanted in Pukou on June 12 during the 2018 and 2019 growing seasons, with harvests conducted on October 22, 2018 and November 12, 2019, respectively. In Liuhe, the single cultivar Nanjing 5055 was transplanted on June 25, 2019 and harvested on November 13, 2019, maintaining consistent phenological scheduling across sites.

### 2.2 Data collection

#### 2.2.1 Determination of LNC

At the booting, flowering, and filling stages, three representative hill plants per plot were destructively sampled from both experimental sites. All leaf samples were immediately transported to the laboratory, where they were oven-dried at 80°C until constant weight was achieved. The dried samples were then finely ground using a stainless-steel mill to ensure homogeneity. LNC was quantified via the micro-Kjeldahl method, which involves sulfuric acid digestion followed by steam distillation and titration, adhering to standardized protocols for agricultural plant tissue analysis.

#### 2.2.2 Image acquisition

**Figure 2:**
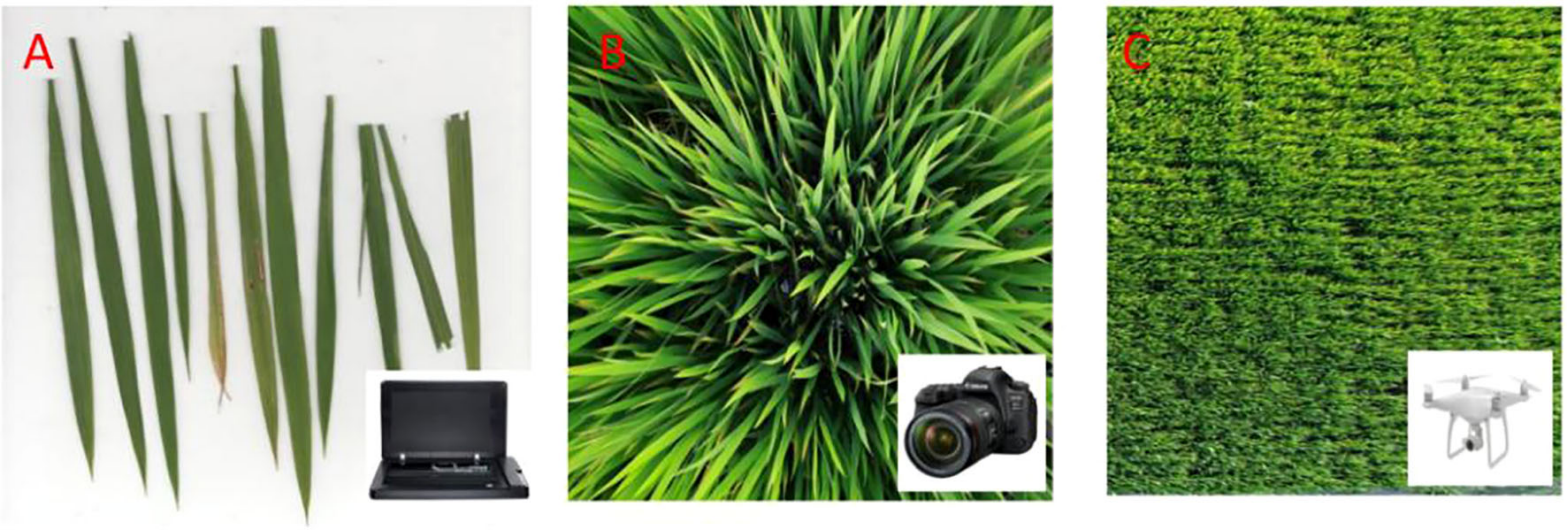
Schematic images from different spatial resolutions: **(A)** leaf-scale image acquired via flatbed scanner (BenQ M209 Pro); **(B)** canopy-scale photograph captured using digital SLR camera (Canon EOS 6D Mark II); **(C)** plot-scale aerial imagery obtained from UAV-mounted consumer-grade camera (DJI Phantom 4 Pro).

Image acquisition was synchronized with destructive leaf sampling at the booting, flowering, and filling stages across both experimental sites (**Table 2**). At the leaf scale, freshly collected samples from each plot were scanned using a BenQ M209 Pro flatbed scanner (BenQ, Inc.) under laboratory conditions. Leaves were horizontally positioned on the scanner platform (297 mm×431.8 mm imaging area) and captured under uniform LED illumination (**Figure 2**). For canopy-scale imaging, a Canon EOS 6D Mark II digital camera (Canon, Inc.) was employed between 11:00 AM and 1:00 PM under stable ambient light, utilizing automated settings for focus, exposure, and white balance. Plot-scale data were acquired via a DJI Phantom 4 Pro UAV (SZ DJI Technology Co., Ltd) equipped with a consumer-grade RGB camera. Three autonomous flights at 50 m, 100 m, and 150 m altitudes maintained a speed of 2 m/s, with 75% lateral and 85% forward overlap to ensure seamless coverage. The camera operated in shutter priority mode (1/1000 s exposure, 2-second intervals), capturing images in JPG format (approximately 20 megapixels) under consistent midday lighting. All RGB images were geotagged and stored in standardized folders for subsequent analysis.

**Table 2:** Images acquisition from leaf, canopy and plot scales in 2018 and 2019.

Experimental site	Year	Spatial scale		
		Leaf scale	Canopy scale	Plot scale
Pukou	2018	✓	×	✓
	2019	✓	✓	✓
Liuhe	2019	✓	✓	✓

### 2.3 Image processing

#### 2.3.1 Image segment

A hierarchical image segmentation workflow was implemented across leaf, canopy, and plot scales to address distinct background complexities. At the leaf scale, images acquired by the BenQ M209 Pro flatbed scanner exhibited uniform white backgrounds (**Figure 2A**). Otsu’s thresholding algorithm—a method maximizing inter-class variance between foreground (leaf) and background (white) pixel intensities—was applied for automated binarization, leveraging the bimodal histogram characteristic of these high-contrast images.

**Figure 3:**
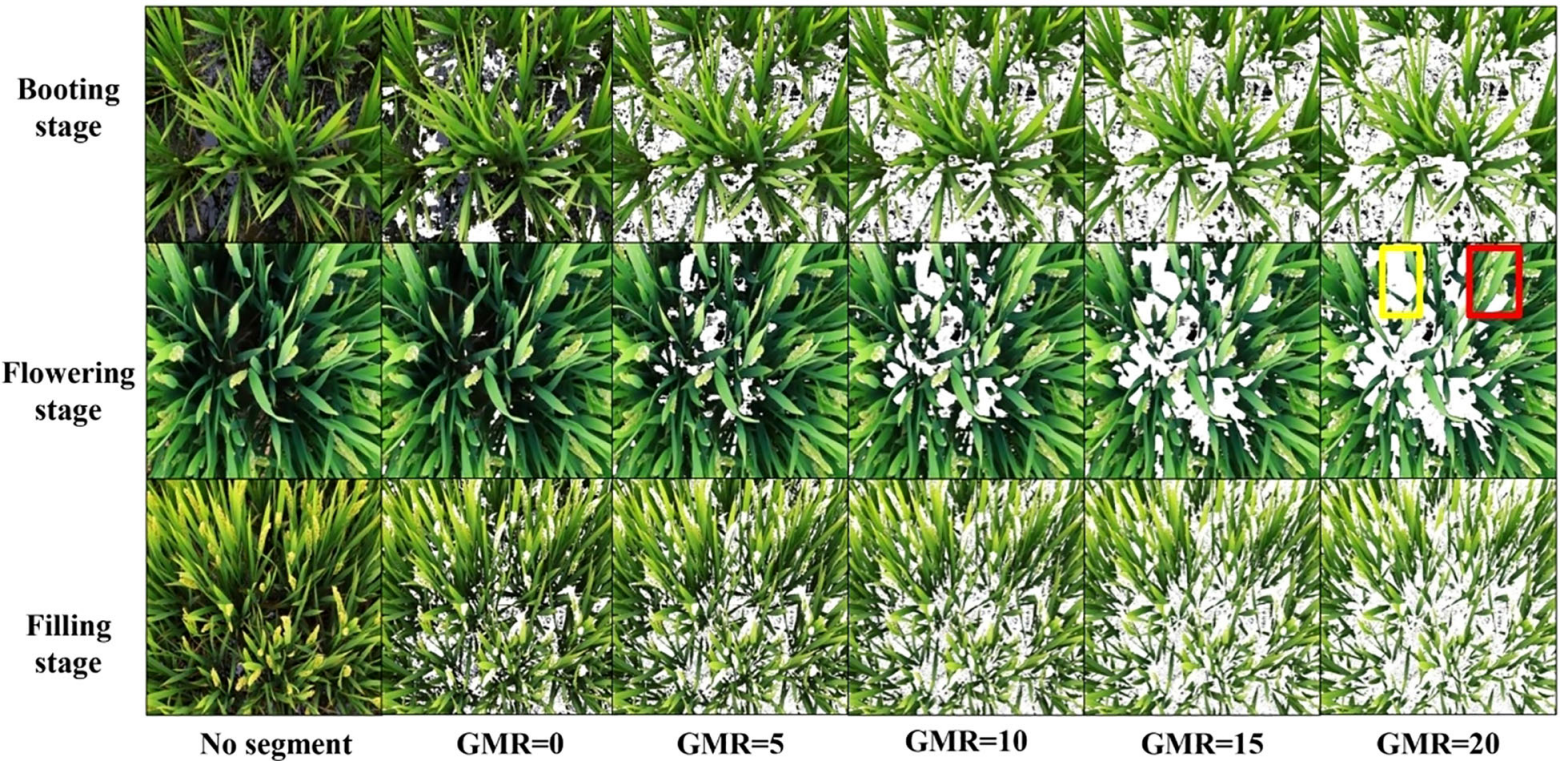
Digital images of rice at canopy scale before and after GMR threshold segmentation. Yellow rectangle indicates the background post-segmentation, while the red rectangle denotes the panicle portion of the canopy image.

For canopy-scale images captured by the Canon EOS 6D Mark II, which contained heterogeneous backgrounds (soil, water, plant residues), vegetation segmentation was achieved through GMR index analysis (Equation 1) This method subtracted red band (R) values from green band (G) values in RGB images, enhancing vegetation/non-vegetation contrast (Wang et al., 2013). Five candidate thresholds (GMR = 0, 5, 10, 15, 20) were empirically tested to optimize canopy-background separation (**Figure 3**). The GMR index was calculated using the following equation:


(1) GMR=G−R

where G and R represent the green and red band values of the RGB image, respectively. The segmentation was implemented using Python, leveraging libraries such as OpenCV and NumPy for image processing. This approach allowed for the efficient application of the GMR threshold to distinguish between vegetation and non-vegetation areas.

At the plot scale, UAV-acquired RGB images first underwent photogrammetric preprocessing in Agisoft PhotoScan Professional (v1.7.3), including feature-based image alignment, orthorectification for geometric correction, and mosaicking to generate georeferenced composites. Subsequently, the GMR segmentation methodology, identical to canopy-scale processing, was applied to ensure multi-scale consistency. This tiered approach balanced scale-specific challenges (uniform vs. complex backgrounds) while maintaining methodological coherence across spatial resolutions.

#### 2.3.2 Calculation of the color indices

In this study, most of the selected color indices have been extensively studied for estimating leaf chlorophyll content, aboveground biomass, and nitrogen status (Li et al., 2010; Saberioon et al., 2014; Wang et al., 2013; Yue et al., 2019). The R, G, and B channels from the color images were used to calculate thirteen color indices (**Table 3**). At the leaf scale, the computation of color indices involved using the mean values of leaf pixels from segmented images of each plot. At the canopy scale, images from each plot were selected to calculate color indices both before and after applying the GMR threshold segmentation method. For the plot scale, the area of interest within each plot was delineated by clipping the UAV-derived orthomosaic images to the plot boundaries. The color indices were then calculated using the mean values of the pixels within the clipped area for each plot. This approach ensures that the indices are representative of the entire plot, accounting for spatial variability within the area of interest.

**Table 3:** Color indices or bands calculated from RGB images in this study.

Index	Description	Formula	Reference
R	The R band of UAV imagery	DN values of R band	–
G	The G band of UAV imagery	DN values of G band	–
B	The B band of UAV imagery	DN values of B band	–
NRI	Normalized red index	R/(R+G+B)	Liu et al. (2019)
NGI	Normalized green index	G/(R+G+B)	Liu et al. (2019)
NBI	Normalized blue index	B/(R+G+B)	Liu et al. (2019)
ExR	Normalized excess red index	(1.4R-G)/(G+R+B)	Meyer and Neto (2008)
ExG	Normalized excess green index	(2G-R-B)/(G+R+B)	Liu et al. (2019)
G/R	Green red ratio index	G/R	Maimaitijiang et al. (2019)
G/B	Green blue ratio index	G/B	Maimaitijiang et al. (2019)
R/B	Red blue ratio index	R/B	Maimaitijiang et al. (2019)
GMR	Green minus red index	G-R	Wang et al. (2013)
INT	Color intensity index	(R+G+B)/3	Ahmad and Reid (1996)

### 2.4 Stepwise multiple linear regressions

In this study, the SMLR technique was employed to establish a correlation between color indices (predictor variables) and LNC (response variable). The SMLR method integrates both forward selection and backward elimination processes to optimize the model. Initially, the forward selection process identifies and includes the predictor variable with the highest statistical significance, provided it meets the 5% significance criterion. At each subsequent step, the process continues to add the most significant remaining variables. Once a new variable is added, the backward elimination process evaluates all included variables, applying a 10% significance criterion to decide whether to remove any variable that no longer meets this threshold. This iterative process of adding and removing variables ensures that only the most statistically significant predictors are retained in the model, enhancing its predictive accuracy and interpretability (Jin et al., 2012).

To further validate the robustness of the SMLR models and mitigate the risk of overfitting, the leave-one-out cross-validation (LOOCV) method was employed. In LOOCV, the model is trained on all data points except one, which is used as the validation set. This process is repeated such that each data point is used once as the validation set. The results are then averaged to provide an estimate of the model’s performance, ensuring that the SMLR model maintains high generalization capability on unseen data. Additionally, to assess the model’s transferability across different sites, cross-site validation was performed. Models were trained on data from the Pukou site and tested on the independent Liuhe site.

### 2.5 Statistical analysis

The correlations between measured LNC and color indices at different growth stages were analyzed using SAS software (version 9.2, SAS Institute Inc., Cary, NC, USA). Model fitness was evaluated by comparing estimated and measured LNC values in a 1:1 plot. The performance of SMLR models for LNC estimation was assessed using the coefficient of determination (R^2^), root mean square error (RMSE), and normalized root mean square error (NRMSE). These statistical indicators were defined in Equations 2–4, respectively, as follows:


(2) $${\text{R}}^{2}=1-\sum_{i=1}^{n}{\left({\text{P}}_{i}-{\text{O}}_{i}\right)}^{2}∕\sum_{i=1}^{n}{\left({\text{P}}_{i}-\bar{\text{O}}\right)}^{2}$$

(3) $$\text{RMSE}=100\times \sqrt{\frac{1}{\text{n}}\times \sum_{i=1}^{n}{\left({\text{P}}_{i}-{\text{O}}_{i}\right)}^{2}}$$

(4) $$\text{NRMSE}=\frac{100}{\bar{\text{O}}}\times \sqrt{\frac{1}{\text{n}}\times \sum_{i=1}^{n}{\left({\text{P}}_{i}-{\text{O}}_{i}\right)}^{2}}$$

where n represents the number of observations, $\bar{O}$ denotes the average value of measured LNC, ${\text{P}}_{i}$ and ${\text{O}}_{i}$ are the estimated and observed values of LNC, respectively. Typically, the simulation is classified as excellent when NRMSE is less than 10%, good if NRMSE is between 10% and 20%, fair if NRMSE is between 20% and 30%, and poor if NRMSE exceeds 30%.

## 3 Results

### 3.1 Variation of measured LNC

The measured LNC across growth stages, experimental sites, years, and nitrogen treatments exhibited substantial variability, ranging from 0.93% to 3.95% (**Table 4**). Consistent with the nitrogen dilution effect, mean LNC declined progressively from booting to filling stages at both sites. At Pukou, mean LNC decreased by 14.3% from 2.31% at the booting stage to 2.01% at the flowering stage, followed by a further 15.4% reduction to 1.71% at the filling stage. Liuhe displayed higher baseline LNC values than Pukou at equivalent phenological phases, with a 15.0% decline from 3.00% at booting to 2.55% at flowering and a 22.4% decrease to 1.98% at filling. This site-specific disparity likely reflects differences in soil fertility, as Liuhe soils contained 19.3% higher organic matter and 23.1% more total nitrogen compared to Pukou.

**Table 4:** Basic information of measured LNC (%) at two experimental sites across various years and growth stages.

Experimental site	n	Booting stage			Flowering stage			Filling stage		
		Min	Max	Mean ± SD	Min	Max	Mean ± SD	Min	Max	Mean ± SD
Pukou	40	1.49	2.96	2.31 ± 0.41	1.25	2.67	2.01 ± 0.35	0.93	2.5	1.71 ± 0.42
Liuhe	30	2.11	3.95	3.00 ± 0.49	1.88	3.39	2.55 ± 0.39	1.21	3.12	1.98 ± 0.50

### 3.2 Estimating LNC from the leaf scale

**Figure 4:**
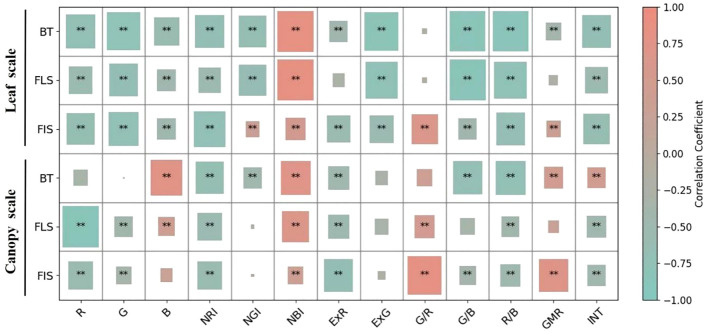
Correlations between color indices and LNC at the leaf and canopy scales before GMR segmentation across growth stages. “BT”, “FLS”, “FIS”, and “All” denote the booting, flowering, grain-filling, and combined stages, respectively. ** indicates significance at p < 0.01.

To assess the efficacy of 13 color indices derived from the leaf scale, correlations between LNC and color indices across various growth stages were established. At the booting stage, nearly all color indices demonstrated significant correlations (p-value< 0.05) with LNC, with the exception of G/R (**Figure 4**). The NBI index exhibited the highest positive correlation with LNC, achieving a coefficient of 0.86^**^, while other color indices showed negative correlations (**Supplementary Table 1**). At the flowering stage, color indices displayed slightly lower correlations with LNC than those observed at the booting stage. NBI and G/B exhibited the highest positive and negative correlations with LNC, achieving coefficients of 0.86^**^ and -0.86^**^, respectively. Furthermore, G/R and GMR demonstrated no significant correlations with LNC. At the filling stage, NRI revealed the highest correlation with LNC, achieving a coefficient of -0.75^**^. Additionally, all color indices demonstrated significant correlations with LNC in rice at the filling stage.

**Figure 5:**
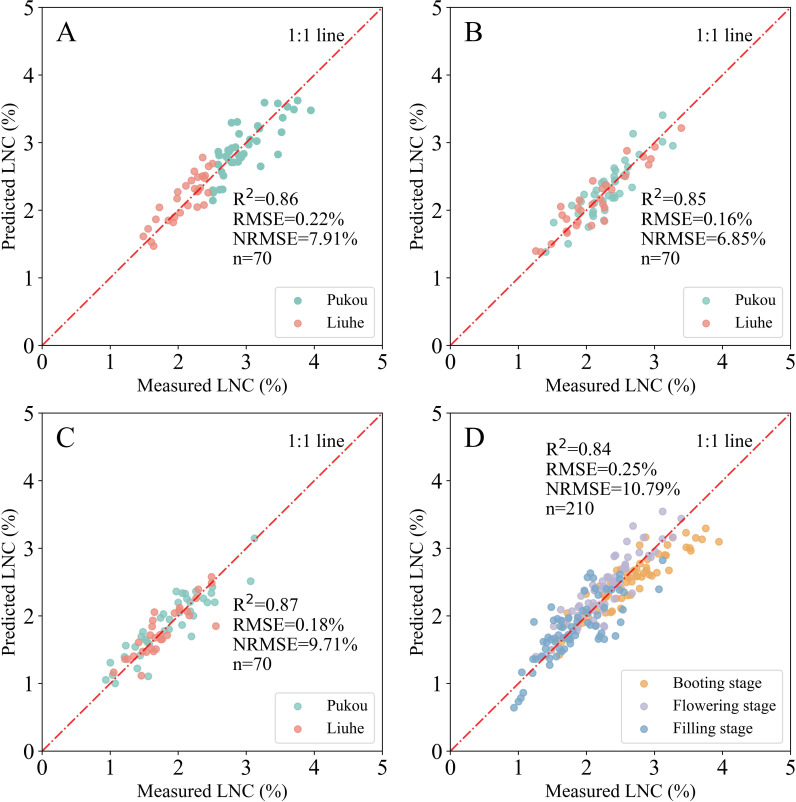
Relationship between measured and predicted LNC using SMLR models at the leaf scale across different growth stages: **(A)** booting, **(B)** flowering, **(C)** filling, and **(D)** combined stages.

Subsequently, these color indices were utilized to construct SMLR models for LNC estimation, as described in Section 2.4. The selected SMLR models at the leaf scale demonstrated remarkable performance across both single-stage and combined-stages datasets. Statistical indicators such as R², RMSE, and NRMSE ranged from 0.84 to 0.87, 0.16% to 0.25%, and 6.85% to 10.79%, respectively, across various growth stages (**Figure 5**).

### 3.3 Estimating LNC from the canopy scale

#### 3.3.1 Estimation of LNC from the canopy scale before image segmentation

Correlations of color indices derived from canopy images with LNC across various growth stages were assessed before image segmentation (**Figure 4**; **Supplementary Table 2**). The highest correlation coefficients were observed for B, R, and G/R indices, achieving 0.75^**^, -0.87^**^ and 0.81^**^, respectively at the booting, flowering, and filling stages. None of the selected optimal color indices exhibited consistent performance in their correlation with LNC across various growth stages.

**Figure 6:**
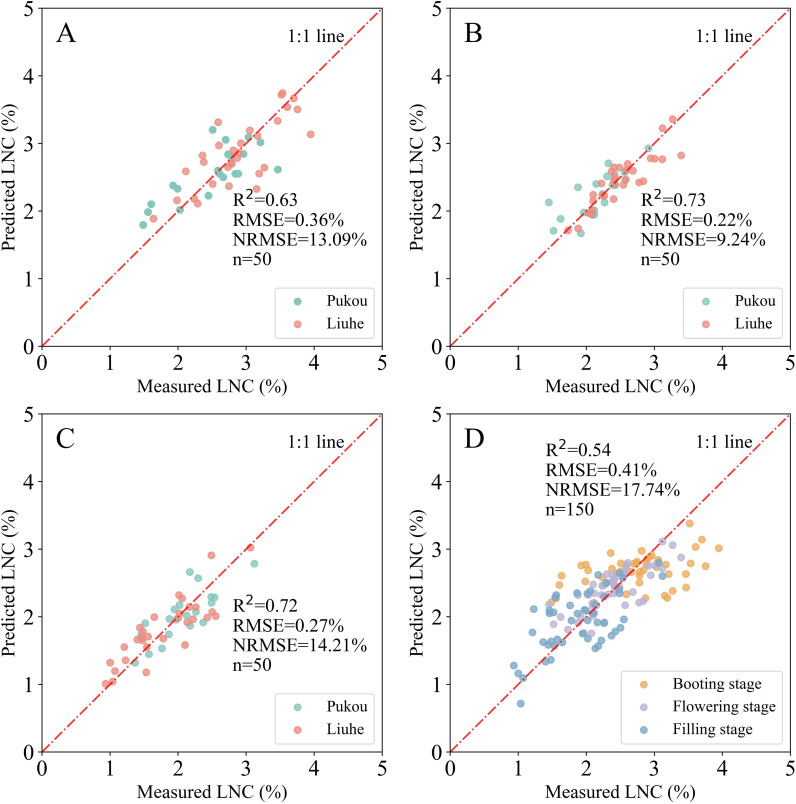
Relationship between measured and predicted LNC using SMLR models at the canopy scale before GMR segmentation across different growth stages: **(A)** booting, **(B)** flowering, **(C)** filling, and **(D)** combined stages.

Among the SMLR models to estimate LNC for single-stage and combined-stages datasets, the SMLR model at combined stages had the poorest performance (R^2^ = 0.54, RMSE = 0.41% and NRMSE = 17.74%) (**Figure 6D**). At the booting stage, the SMLR model overestimated LNC for values lower than 2% and underestimated it for values higher than 3.6% (**Figure 6A**, data not shown). The performances of the SMLR models at the flowering and filling stages were similar, with R^2^ values of 0.73 and 0.72, RMSE values of 0.22% and 0.27%, and NRMSE values of 9.24% and 14.21%, respectively (**Figure 6**).

#### 3.3.2 Estimation of LNC from the canopy scale after image segmentation

**Figure 7:**
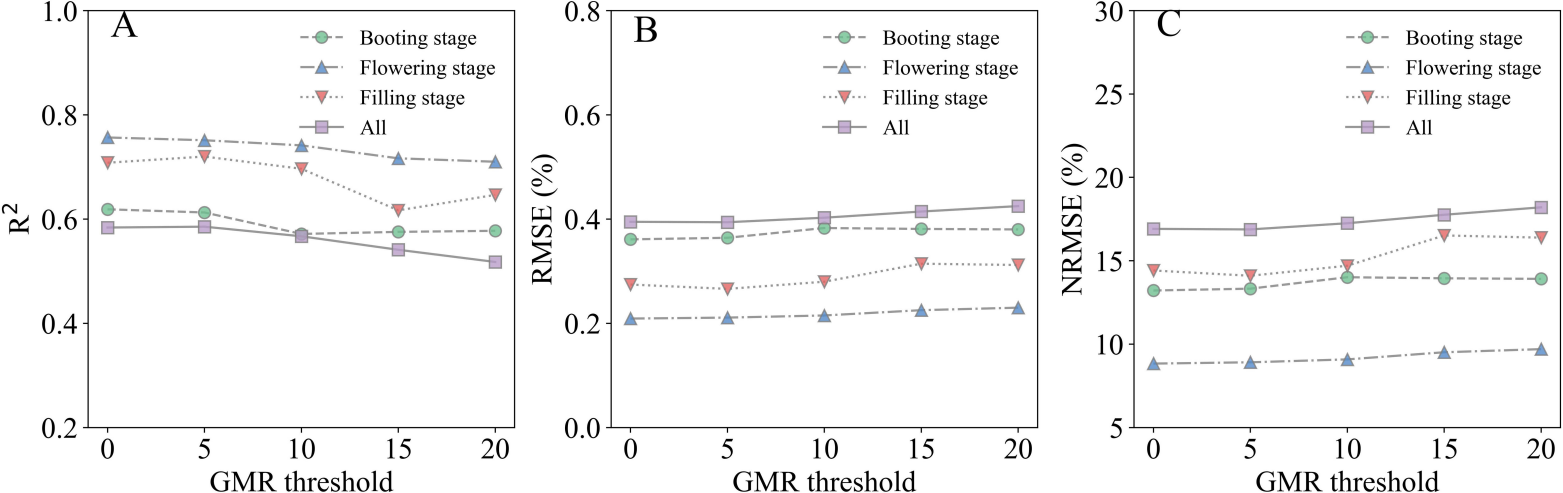
Performance of SMLR models in estimating rice LNC at the canopy scale following GMR threshold segmentation. **(A)** R^2^, **(B)** RMSE, **(C)** NRMSE.

The performance of SMLR models derived from canopy-scale images after GMR threshold segmentation is illustrated in **Figure 7**. To compare the robustness and reliability of the models across varying GMR threshold values, these were set from 0 to 20 in increments of 5. Generally, the R^2^ values exhibited similar trends across various GMR threshold values, with the exception of a GMR value of 20 at the filling stage. R^2^ values tended to decrease as GMR threshold values increased, both in single-stage and combined-stages datasets. When GMR threshold values exceeded 5, both RMSE and NRMSE tended to increase with higher GMR thresholds. Following image segmentation, the SMLR model at the flowering stage showed optimal performance for estimating LNC, both in single-stage and combined-stages datasets, at consistent GMR threshold values. This result parallels the performance observed with the SMLR model at the flowering stage before image segmentation (**Figure 6B**).

### 3.4 Estimating LNC from the plot scale

#### 3.4.1 Performance of SMLR models across flight altitudes (pre-segmentation)

Correlation analysis between LNC and UAV-derived color indices revealed altitude-dependent patterns (**Supplementary Table 3**). At booting and flowering stages, the NRI exhibited the strongest negative correlations with LNC across most altitudes (|r| = 0.66–0.79), except at 50 m during booting. At the filling stage, optimal indices shifted: ExR dominated at 50 m and 150 m, while the R band showed peak correlation at 100 m.

The SMLR models demonstrated distinct altitude-dependent performance in estimating rice LNC from UAV-based RGB imagery (**Table 5**). At 100 m flight altitude, models consistently achieved optimal accuracy across growth stages, with R^2^ values ranging from 0.61 (filling stage) to 0.65 (booting stage), RMSE between 0.29% (flowering) and 0.35% (all stages), and NRMSE of 13%-16%. This altitude outperformed both lower (50 m) and higher (150 m) alternatives—for instance, at the booting stage, 100 m altitude improved R^2^ by 3.2% compared to 50 m and 8.3% over 150 m. The exception occurred during flowering stage, where 150 m altitude unexpectedly showed marginally better NRMSE (12% vs. 13% at 100 m), though with comparable R^2^ (0.63 vs. 0.61). Notably, combined-stage models at 100 m altitude maintained robust performance (R^2^ = 0.65, NRMSE = 16%), suggesting stable generalizability across phenological phases. The superior performance at 100 m likely stems from an optimal balance between spatial resolution and reduced atmospheric interference, enabling precise vegetation indexing while minimizing sensor noise. These findings establish 100 m as the recommended operational altitude for plot-scale LNC monitoring via consumer-grade UAVs in rice paddies.

**Table 5:** Performance metrics of SMLR models for LNC estimation across flight altitudes and growth stages (pre-segmentation).

Growth stage	Flight altitude								
	50 m			100 m			150 m		
	R^2^	RMSE	NRMSE	R^2^	RMSE	NRMSE	R^2^	RMSE	NRMSE
Booting stage	0.63	0.34%	13%	0.65	0.33%	13%	0.60	0.36%	14%
Flowering stage	0.57	0.30%	13%	0.61	0.29%	13%	0.63	0.28%	12%
Filling stage	0.58	0.31%	17%	0.61	0.29%	16%	0.57	0.31%	17%
All	0.61	0.37%	16%	0.65	0.35%	16%	0.63	0.36%	16%

#### 3.4.2 Performance of the SMLR models obtained from different flight altitudes after image segmentation

**Figure 8:**
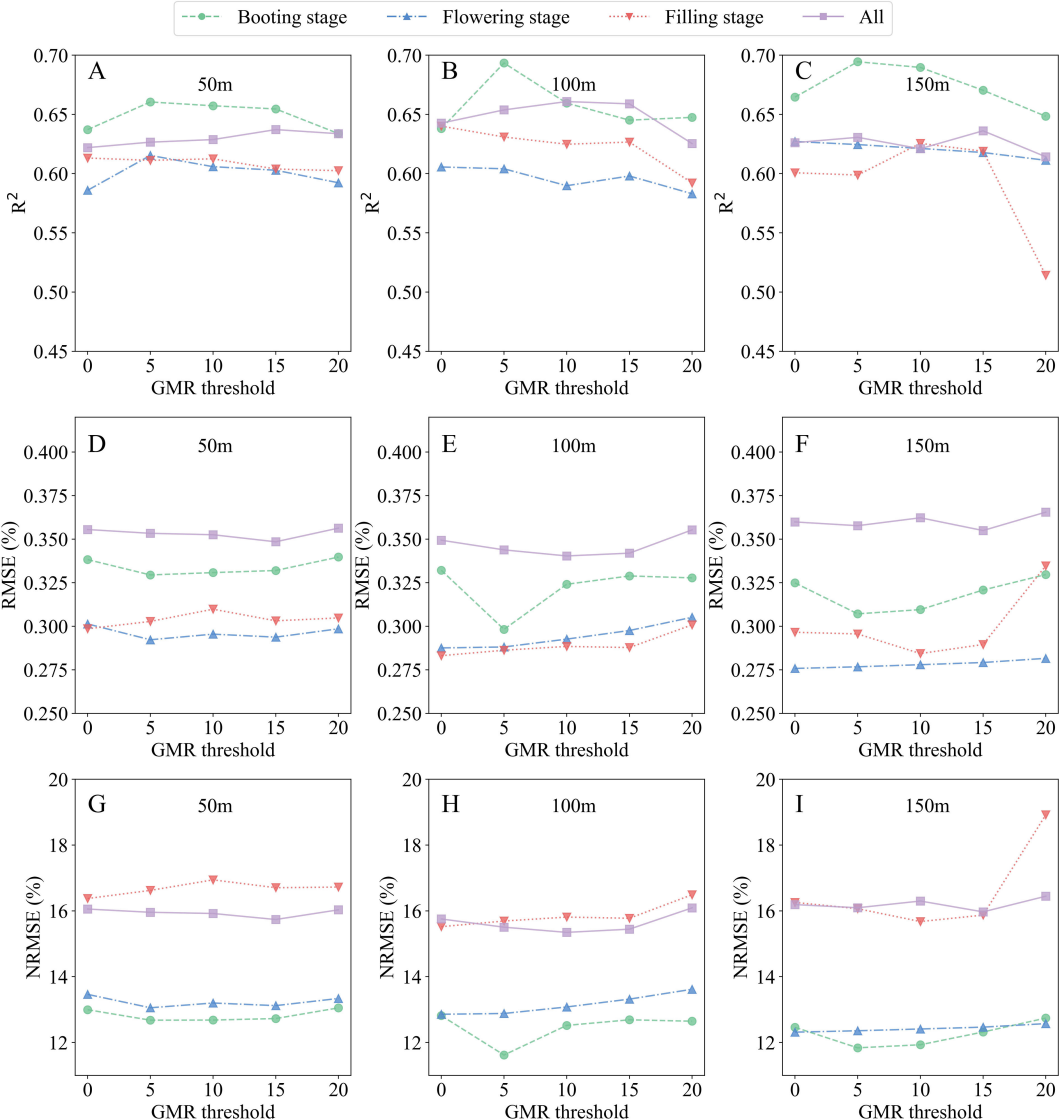
Performance of the selected SMLR models for estimating LNC at the plot scale after image segmentation. **(A, D, G)** R^2^, RMSE, and NRMSE at 50-m flight altitude; **(B, E, H)** R^2^, RMSE, and NRMSE at 100-m flight altitude; **(C, F, I)** R^2^, RMSE, and NRMSE at 150-m flight altitude.

Post-segmentation analysis of SMLR models across flight altitudes revealed nuanced performance patterns tied to GMR threshold selection (**Figure 8**). At 50 m altitude, R^2^ values exhibited a unimodal trend with increasing GMR thresholds (0–20), peaking at intermediate thresholds before declining in both single-stage and combined datasets. This pattern corresponded to minimal variation in RMSE and NRMSE (coefficient of variation< 1.5%, **Supplementary Table 4**). At an altitude of 100 m, model performance demonstrated stability across different thresholds. Single-stage datasets yielded R^2^ ranging from 0.58 to 0.66, with RMSE of 0.29% to 0.36% and NRMSE of 11.6% to 16.5%. Combined-stage datasets showed R^2^ values of 0.63 to 0.66, RMSE values of 0.34% to 0.36%, and NRMSE values of 15.4% to 16.1%. At 150 m altitude, LNC estimation accuracy deteriorated sharply when GMR thresholds exceeded 10, except in the combined-stage dataset, mirroring trends observed at 100 m but with lower robustness.

Statistical variability across thresholds remained moderate, with coefficients of variation ranging from 0.83% to 7.8% for R^2^, 0.8% to 6.6% for RMSE, and 0.79% to 8.08% for NRMSE—confirming the tested thresholds exhibited minimal sensitivity within the evaluated range. Post-segmentation performance peaked consistently at 100 m altitude, where models achieved a balanced trade-off between precision (R^2^ up to 0.66) and error minimization (NRMSE 10%–20%), validating the GMR segmentation framework detailed in Section 2.4. These findings establish 100 m as the most operationally suitable altitude for plot-scale LNC monitoring using UAV-derived RGB imagery.

### 3.5 Model transferability across sites

To evaluate model generalizability across spatial heterogeneities, cross-site validation was performed using models trained on Pukou site data and tested on the independent Liuhe site. Performance metrics are stratified by spatial scale (leaf, canopy, plot) and flight altitude (for plot-scale models: 50 m, 100 m, 150 m), as summarized in **Supplementary Table 5**.

For the leaf scale, Pukou-trained models showed strong cross-site performance, with training R^2^ of 0.72, RMSE of 0.32%, and NRMSE of 12.3%. Test results were R^2^ of 0.59, RMSE of 0.37%, and NRMSE of 16.8%, demonstrating robustness against inter-site spectral variability. At the canopy scale, transferability declined: training yielded R^2^ of 0.63, RMSE of 0.34%, and NRMSE of 15.1%, while test values dropped to R^2^ of 0.45, RMSE of 0.48%, and NRMSE of 19.8%. Plot-scale performance proved altitude-sensitive: at 50 m, test R^2^ was 0.40 with RMSE 0.47% and NRMSE 21.4%. Slight improvements were observed at 100 m (R^2^ = 0.39, RMSE = 0.46%, NRMSE = 21.1%), followed by a marginal decline at 150 m (R^2^ = 0.38, RMSE = 0.46%, NRMSE = 20.9%), likely due to reduced spatial resolution limiting fine-scale nitrogen detection.

## 4 Discussion

### 4.1 Effect of spatial scale on LNC estimation accuracy

**Figure 9:**
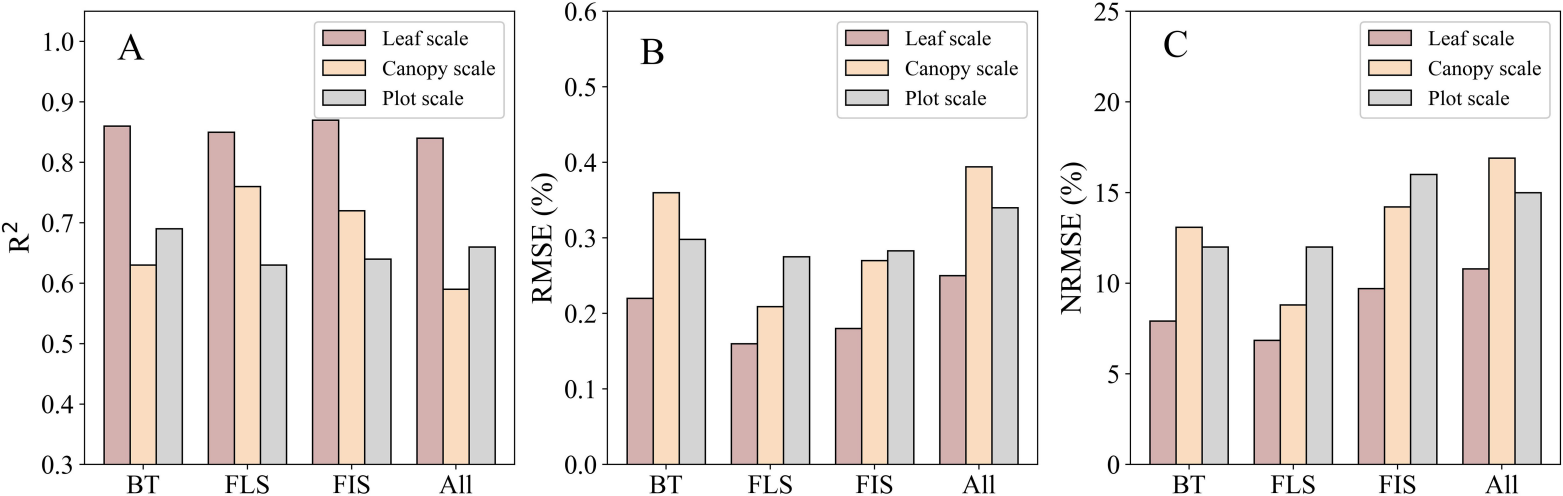
Comparison analysis between the optimal SMLR models from leaf, canopy and plot scales. “BT”, “FLS”, “FIS”, and “All” represent the booting, flowering, filling, and combined stages, respectively. **(A)** R^2^, **(B)** RMSE, **(C)** NRMSE.

This study highlights the critical role of spatial scale in determining the efficacy of RGB imaging for rice LNC assessment. At the leaf scale, the SMLR models achieved superior accuracy (R^2^ = 0.84–0.87) compared to canopy- and plot-scale approaches (**Figure 9**). This advantage stemmed from stringent laboratory conditions: leaves were flattened against the scanner surface, ensuring uniform orientation and lighting, while the white background facilitated near-perfect segmentation via Otsu’s method. By eliminating field variability (e.g., shadows, soil interference), leaf-scale imaging isolated spectral responses directly linked to nitrogen content. These findings align with Saberioon et al. (2014), who demonstrated that controlled leaf-scale imaging enables rapid nitrogen diagnostics, though their focus on single leaves contrasts with our multi-stage integration. The cross-site validation (Section 3.5) further supported the leaf-scale model’s generalizability, showing relatively stable performance when applied to a different site. This indicates that the leaf-scale model can capture fundamental spectral-nitrogen relationships that persist across environments, making it promising for broader applications.

At the​canopy scale, the high-resolution DSLR camera (26 megapixels) captured intricate canopy details but introduced noise from heterogeneous backgrounds (soil, water residues). Model performance exhibited phenological dependency: flowering and filling stages achieved higher accuracy (R^2^ improvement > 15% vs. booting stage), likely due to stabilized canopy architecture. During booting, dense foliage and vertical leaf angles increased intra-canopy shadowing, reducing spectral discriminability. This aligns with Bendig et al. (2015), who reported similar structural complexity challenges in barley biomass estimation. Additionally, visible reflectance saturation at booting stage—attributable to high leaf area index (LAI > 4.5) and chlorophyll density—limited model sensitivity, as observed in rice nitrogen accumulation studies (Zheng et al., 2018). Such saturation phenomena are well-documented in crops with overlapping leaves, underscoring the need for phenology-specific calibration.

Plot-scale UAV imaging faced unique tradeoffs between spatial resolution and environmental interference. At 50 m altitude, bidirectional reflectance under solar noon conditions necessitated intensive image stitching to mitigate angular artifacts. Conversely, 150 m altitude introduced motion blur and chromatic aberrations in consumer-grade cameras, degrading spectral fidelity. The 100 m altitude optimally balanced resolution and stability, yielding consistent accuracy (NRMSE< 16%). These observations corroborate Rasmussen et al. (2016), who identified solar angle variations as a key uncertainty in UAV-based vegetation indexing. Post-segmentation GMR thresholding further enhanced accuracy by suppressing soil/water interference, validating its utility in operational settings.

Collectively, these results establish a scale-dependent hierarchy: leaf-scale precision, canopy-scale field adaptability, and plot-scale scalability. Each scale addresses distinct agricultural needs, from high-stakes breeding trials to regional fertilizer management.

### 4.2 Impact of GMR threshold segmentation on LNC estimation

To assess the sensitivity of SMLR models to GMR segmentation thresholds in rice LNC estimation, we systematically evaluated threshold values from 0 to 20 in 5-unit increments. This analysis excluded leaf-scale imagery due to its uniform white background, which permits reliable segmentation via Otsu’s method without requiring advanced techniques. Wang et al. (2013) established the efficacy of GMR segmentation for rice-background isolation in digital imagery, providing a methodological foundation for our canopy- and plot-scale analyses.

At the canopy scale, GMR thresholds > 5 during the booting stage effectively differentiated rice canopies from complex backgrounds (soil, water residues) in intact images (**Figure 3**). Paradoxically, post-segmentation SMLR models exhibited reduced accuracy compared to pre-segmentation models (**Table 6**), suggesting that canopy-level segmentation may filter ecologically relevant spectral variation. This observation aligns with Lee and Lee (2013), who demonstrated stronger correlations between VIs and crop parameters in non-segmented datasets, implying that segmentation disrupts natural reflectance gradients associated with canopy density and microtopography. The challenge intensified during flowering and filling stages, where panicles and senescing leaves exhibited spectral similarities (**Figure 3**), complicating pixel classification. However, combined-stage models demonstrated notable accuracy improvements post-segmentation (R^2^ increase ≤ 9.26%, **Table 6**), likely because multi-temporal calibration compensates for illumination variations and structural heterogeneity. This compensation mechanism aligns with the principle that temporal integration buffers single-stage segmentation errors (Zheng et al., 2018).

**Table 6:** The statistic of the improved accuracy (R^2^) of the optimal SMLR models after GMR threshold segmentation method at canopy and plot scales.

Spatial scales	Altitudes above canopy	Growth stage			
		Booting stage	Flowering stage	Filling stage	All
Canopy scale	1m	-1.59%	4.11%	0.00%	9.26%
Plot scale	50m	4.76%	8.77%	5.17%	4.92%
	100m	6.15%	0.00%	4.92%	1.54%
	150m	15%	0.00%	10.53%	1.59%

At the plot scale, it is notable that most researchers have not incorporated UAV-based image segmentation into their models for crop parameter estimation (Li et al., 2016). This oversight may lead to inaccuracies in parameter estimation. In this study, we adopted specific GMR threshold values to segment images obtained from different flight altitudes, aiming to enhance the accuracy of LNC estimation. This approach allowed us to address the variations in lighting and perspective associated with different altitudes. During the reproductive stage, rice achieves nearly full coverage, creating significant shading effects. The darker regions in the plot images were primarily due to the lower parts of the rice canopy being shaded by the upper parts (Wang et al., 2013). This shading effect poses challenges for accurate image segmentation and analysis. When the GMR threshold value exceeded 0, darker areas were classified as background. After segmentation, the optimal SMLR models showed some enhancement in the reliability of the assessments (**Table 6**), indicating the potential benefits of segmentation in improving model accuracy. However, ANOVA revealed no significant differences in the R^2^ values obtained from various altitudes at the same growth stage after GMR segmentation (p > 0.05). This suggests that while segmentation can enhance model reliability, its impact may be limited by other factors such as altitude and growth stage.

In summary, our findings indicate that while GMR segmentation can enhance the reliability of SMLR models for LNC estimation, its effectiveness varies across different spatial scales and growth stages. This variability underscores the need for tailored segmentation approaches when monitoring rice nitrogen status.

## 5 Conclusions

In this study, the performance of SMLR models in estimating the LNC of rice was investigated using color digital images from three different spatial scales. The results indicated that SMLR models at the leaf scale demonstrated the most robust predictive abilities for LNC estimation across growth stages when compared to other spatial scales. At the canopy scale, SMLR models exhibited acceptable precision and accuracy in LNC estimation prior to image segmentation. Further research indicated that accuracy improvements were more significant in combined-stage datasets than in single-stage datasets following GMR segmentation. At the plot scale, the impact of images derived from UAVs at different flight altitudes on LNC estimation was analyzed both before and after GMR threshold segmentation. Generally, UAV images acquired at a flight altitude of 100 m showed relatively suitable performance for estimating rice LNC, applicable to both single and combined growth stages. Cross-site validation revealed that the leaf-scale model maintained strong performance when applied to an independent site, while the canopy and plot-scale models showed greater sensitivity to site-specific conditions. These findings underscore the importance of considering the effects of different spatial scales and environmental conditions when developing models for assessing crop biophysical and biochemical parameters. This information will also be useful for selecting sensors and designing improved sensors for ecosystem observation. Moreover, it highlights the significant advantages and potential of using threshold segmentation techniques in digital images to enhance the accuracy of crop nitrogen monitoring.

## Data Availability

Inquiries regarding the datasets presented in this article can be directed to the corresponding authors. Requests to access the datasets should be directed to Haixiao Ge gehx@jsou.edu.cn.

## References

[B1] Ahmad I. S. Reid J. F. (1996). Evaluation of colour representations for maize images. J. Agric. Eng. Res. 63, 185–195. doi: 10.1006/jaer.1996.0020

[B2] Bendig J. Yu K. Aasen H. Bolten A. Bennertz S. Broscheit J.. (2015). Combining UAV-based plant height from crop surface models, visible, and near infrared vegetation indices for biomass monitoring in barley. Int. J. Appl. Earth Obs. Geoinf. 39, 79–87. doi: 10.1016/j.jag.2015.02.012

[B3] Crema A. Boschetti M. Nutini F. Cillis D. Casa R. (2020). Influence of soil properties on maize and wheat nitrogen status assessment from sentinel-2 data. Remote Sens. 12, 2175. doi: 10.3390/rs12142175

[B4] Dai C. Sun J. Huang X. Zhang X. Tian X. Wang W.. (2023). Application of hyperspectral imaging as a nondestructive technology for identifying tomato maturity and quantitatively predicting lycopene content. Foods 12, 2957. doi: 10.3390/foods12152957, PMID: 37569225 PMC10418690

[B5] Erdle K. Mistele B. Schmidhalter U. (2011). Comparison of active and passive spectral sensors in discriminating biomass parameters and nitrogen status in wheat cultivars. Field Crops Res. 124, 74–84. doi: 10.1016/j.fcr.2011.06.012

[B6] Feng W. Guo B.-B. Wang Z.-J. He L. Song X. Wang Y.-H.. (2014). Measuring leaf nitrogen concentration in winter wheat using double-peak spectral reflection remote sensing data. Field Crops Res. 159, 43–52. doi: 10.1016/j.fcr.2014.01.007

[B7] He L. Zhang H. Y. Zhang Y. S. Song X. Feng W. Kang G. Z.. (2016). Estimating canopy leaf nitrogen concentration in winter wheat based on multi-angular hyperspectral remote sensing. Eur. J. Agron. 73, 73–83. doi: 10.1016/j.eja.2015.11.011

[B8] Jin X. L. Wang K. R. Xiao C. H. Diao W. Y. Wang F. Y. Chen B.. (2012). Comparison of two methods for estimation of leaf total chlorophyll content using remote sensing in wheat. Field Crops Res. 135, 21–28. doi: 10.1016/j.fcr.2012.07.002

[B9] Kalacska M. Lalonde M. Moore T. R. (2015). Estimation of foliar chlorophyll and nitrogen content in an ombrotrophic bog from hyperspectral data: Scaling from leaf to image. Remote Sens. Environ. 169, 270–279. doi: 10.1016/j.rse.2015.08.012

[B10] Lee K.-J. Lee B.-W. (2013). Estimation of rice growth and nitrogen nutrition status using color digital camera image analysis. Eur. J. Agron. 48, 57–65. doi: 10.1016/j.eja.2013.02.011

[B11] Lepine L. C. Ollinger S. V. Ouimette A. P. Martin M. E. (2016). Examining spectral reflectance features related to foliar nitrogen in forests: Implications for broad-scale nitrogen mapping. Remote Sens. Environ. 173, 174–186. doi: 10.1016/j.rse.2015.11.028

[B12] Li Y. Chen D. Walker C. N. Angus J. F. (2010). Estimating the nitrogen status of crops using a digital camera. Field Crops Res. 118, 221–227. doi: 10.1016/j.fcr.2010.05.010

[B13] Li F. Mistele B. Hu Y. Yue X. Yue S. Miao Y.. (2012). Remotely estimating aerial N status of phenologically differing winter wheat cultivars grown in contrasting climatic and geographic zones in China and Germany. Field Crops Res. 138, 21–32. doi: 10.1016/j.fcr.2012.09.014

[B14] Li W. Niu Z. Chen H. Li D. Wu M. Zhao W. (2016). Remote estimation of canopy height and aboveground biomass of maize using high-resolution stereo images from a low-cost unmanned aerial vehicle system. Ecol. Indic. 67, 637–648. doi: 10.1016/j.ecolind.2016.03.036

[B15] Liu K. Li Y. Han T. Yu X. Ye H. Hu H.. (2019). Evaluation of grain yield based on digital images of rice canopy. Plant Methods 15, 28. doi: 10.1186/s13007-019-0498-5, PMID: 30949229 PMC6429754

[B16] Lu J. Cheng D. Geng C. Zhang Z. Xiang Y. Hu T. (2021). Combining plant height, canopy coverage and vegetation index from UAV-based RGB images to estimate leaf nitrogen concentration of summer maize. Biosyst. Eng. 202, 42–54. doi: 10.1016/j.biosystemseng.2020.11.004

[B17] Maimaitijiang M. Sagan V. Sidike P. Maimaitiyiming M. Hartling S. Peterson K. T.. (2019). Vegetation Index Weighted Canopy Volume Model (CVMVI) for soybean biomass estimation from Unmanned Aerial System-based RGB imagery. ISPRS J. Photogramm. Remote Sens. 151, 27–41. doi: 10.1016/j.isprsjprs.2019.03.003

[B18] Meyer G. E. Neto J. C. (2008). Verification of color vegetation indices for automated crop imaging applications. Comput. Electron. Agric. 63, 282–293. doi: 10.1016/j.compag.2008.03.009

[B19] Nirere A. Sun J. Atindana V. A. Hussain A. Zhou X. Yao K. (2022). A comparative analysis of hybrid SVM and LS-SVM classification algorithms to identify dried wolfberry fruits quality based on hyperspectral imaging technology. J. Food Process. Preserv. 46, e16320. doi: 10.1111/jfpp.16320

[B20] Padilla F. M. Teresa Peña-Fleitas M. Gallardo M. Thompson R. B. (2014). Evaluation of optical sensor measurements of canopy reflectance and of leaf flavonols and chlorophyll contents to assess crop nitrogen status of muskmelon. Eur. J. Agron. 58, 39–52. doi: 10.1016/j.eja.2014.04.005

[B21] Rasmussen J. Ntakos G. Nielsen J. Svensgaard J. Christensen S. (2016). Are vegetation indices derived from consumer-grade cameras mounted on UAVs sufficiently reliable for assessing experimental plots? Eur. J. Agron. 74, 75–92. doi: 10.1016/j.eja.2015.11.026

[B22] Saberioon M. M. Amin M. S. M. Anuar A. R. Gholizadeh A. Wayayok A. Khairunniza-Bejo S. (2014). Assessment of rice leaf chlorophyll content using visible bands at different growth stages at both the leaf and canopy scale. Int. J. Appl. Earth Obs. Geoinf. 32, 35–45. doi: 10.1016/j.jag.2014.03.018

[B23] Shi P. Wang Y. Xu J. Zhao Y. Yang B. Yuan Z.. (2021). Rice nitrogen nutrition estimation with RGB images and machine learning methods. Comput. Electron. Agric. 180, 105860. doi: 10.1016/j.compag.2020.105860

[B24] Shukla A. K. Ladha J. K. Singh V. K. Dwivedi B. S. Vethaiya B. Gupta R. K.. (2004). Calibrating the leaf color chart for nitrogen management in different genotypes of rice and wheat in a systems perspective. Agron. J. 96, 1606–1621. doi: 10.2134/agronj2004.1606

[B25] Tian Y. Sun J. Zhou X. Yao K. Tang N. (2022). Detection of soluble solid content in apples based on hyperspectral technology combined with deep learning algorithm. J. Food Process. Preserv. 46, e16414. doi: 10.1111/jfpp.16414

[B26] Wang Y. Wang D. Zhang G. Wang J. (2013). Estimating nitrogen status of rice using the image segmentation of G-R thresholding method. Field Crops Res. 149, 33–39. doi: 10.1016/j.fcr.2013.04.014

[B27] Xu X. He W. Zhang H. (2024). Random hierarchical model for estimation of wheat yield in the North China Plain at different spatial scales. Field Crops Res. 306, 109241. doi: 10.1016/j.fcr.2023.109241

[B28] Xu M. Sun J. Cheng J. Yao K. Wu X. Zhou X. (2023). Non-destructive prediction of total soluble solids and titratable acidity in Kyoho grape using hyperspectral imaging and deep learning algorithm. Int. J. Food Sci. Technol. 58, 9–21. doi: 10.1111/ijfs.16186

[B29] Yue J. Yang G. Tian Q. Feng H. Xu K. Zhou C. (2019). Estimate of winter-wheat above-ground biomass based on UAV ultrahigh-ground-resolution image textures and vegetation indices. ISPRS J. Photogramm. Remote Sens. 150, 226–244. doi: 10.1016/j.isprsjprs.2019.02.022

[B30] Zhang Z. Xu X. Jin M. Bai J. Shu X. Deng L.. (2024). A new curve of critical leaf nitrogen concentration based on the maximum root dry matter for diagnosing nitrogen nutritional status of sweetpotato. Eur. J. Agron. 156, 127143. doi: 10.1016/j.eja.2023.127143

[B31] Zhang M. Zhou J. Sudduth K. A. Kitchen N. R. (2020). Estimation of maize yield and effects of variable-rate nitrogen application using UAV-based RGB imagery. Biosyst. Eng. 189, 24–35. doi: 10.1016/j.biosystemseng.2019.11.004

[B32] Zheng H. Cheng T. Li D. Zhou X. Yao X. Tian Y.. (2018). Evaluation of RGB, color-infrared and multispectral images acquired from unmanned aerial systems for the estimation of nitrogen accumulation in rice. Remote Sens. 10, 824. doi: 10.3390/rs10060824

