# Supplementary material for: Assessing leaf nitrogen concentration in rice using RGB imaging: a comparative study at leaf, canopy, and plot scales
Source: Front Plant Sci. 2025 Aug 5;16:1599177. doi: 10.3389/fpls.2025.1599177 (PMC12361148; doi:10.3389/fpls.2025.1599177)
Supplement: Supplementary file 1 [file Table1.docx]

Supplementary Material

# Supplementary Table 1

Correlation analysis between LNC and color indices derived from the leaf scale across different growth stages.

| **Color index** | **Growth stage** | | |
| --- | --- | --- | --- |
|  | **Booting stage** | **Flowering stage** | **Filling stage** |
| R | -0.71^**^ | -0.57^**^ | -0.67^**^ |
| G | -0.80^**^ | -0.69^**^ | -0.72^**^ |
| B | -0.60^**^ | -0.45^**^ | -0.45^**^ |
| NRI | -0.69^**^ | -0.53^**^ | **-0.75^**^** |
| NGI | -0.67^**^ | -0.65^**^ | 0.34^**^ |
| NBI | **0.86^**^** | **0.86^**^** | 0.48^**^ |
| ExR | -0.44^**^ | -0.29^*^ | -0.56^**^ |
| ExG | -0.81^**^ | -0.77^**^ | -0.58^**^ |
| G/R | -0.12^n.s.^ | -0.12^n.s.^ | 0.63^**^ |
| G/B | -0.85^**^ | **-0.86^**^** | -0.44^**^ |
| R/B | -0.85^**^ | -0.78^**^ | -0.68^**^ |
| GMR | -0.38^**^ | -0.22^n.s.^ | 0.35^**^ |
| INT | -0.69^**^ | -0.55^**^ | -0.64^**^ |

**Note:** n.s., ^*^, and ^**^ represent ‘not significant’, p<0.05, and p<0.01, respectively.

**Supplementary Table 2**

Correlation analysis between LNC and color indices derived from the canopy scale across different growth stages before image segmentation.

| Color index | Growth stage | | |
| --- | --- | --- | --- |
|  | Booting stage | Flowering stage | Filling stage |
| R | -0.34^*^ | **-0.87^**^** | -0.60^**^ |
| G | 0.04^n.s.^ | -0.43^**^ | -0.37^**^ |
| B | **0.75^**^** | 0.40^**^ | 0.29^*^ |
| NRI | -0.67^**^ | -0.58^**^ | -0.60^**^ |
| NGI | -0.45^**^ | -0.09^n.s.^ | 0.07^n.s.^ |
| NBI | 0.71^**^ | 0.65^**^ | 0.37^**^ |
| ExR | -0.50** | -0.51^**^ | -0.69^**^ |
| ExG | -0.30^*^ | -0.34^*^ | -0.19^n.s.^ |
| G/R | 0.36^*^ | 0.49^**^ | **0.81^**^** |
| G/B | -0.70^**^ | -0.35^*^ | -0.40^**^ |
| R/B | -0.71^**^ | -0.43^**^ | -0.48^**^ |
| GMR | 0.46^**^ | 0.27^n.s.^ | 0.70^**^ |
| INT | 0.43^**^ | -0.46^**^ | -0.45^**^ |

**Note:** n.s., ^*^, and ^**^ represent ‘not significant’, p<0.05, and p<0.01, respectively.

**Supplementary Table 3**

Correlation analysis between LNC and color indices derived from the plot scale across different growth stages before image segmentation.

| Color index | Growth stage | | | | | | | | |
| --- | --- | --- | --- | --- | --- | --- | --- | --- | --- |
|  | Booting stage | | | Flowering stage | | | Filling stage | | |
|  | 50 m | 100 m | 150 m | 50 m | 100 m | 150 m | 50 m | 100 m | 150 m |
| R | -0.59^**^ | -0.57^**^ | 0.24^n.s.^ | -0.48^**^ | -0.66^**^ | 0.21^n.s.^ | -0.66^**^ | **-0.81^**^** | 0.63^**^ |
| G | **-0.72^**^** | -0.75^**^ | 0.12^n.s.^ | -0.39^*^ | -0.61^**^ | 0.14^n.s.^ | -0.51^**^ | -0.79^**^ | 0.46^**^ |
| B | 0.39^**^ | 0.45^**^ | 0.35^*^ | 0.49^**^ | 0.1^n.s.^ | 0.34^*^ | -0.43^**^ | -0.59^**^ | 0.42^**^ |
| NRI | -0.71^**^ | **-0.79^**^** | **-0.75^**^** | **-0.66^**^** | **-0.78^**^** | **-0.76^**^** | -0.65^**^ | -0.57^**^ | 0.69^**^ |
| NGI | -0.42^**^ | -0.44^**^ | -0.28^*^ | -0.3^*^ | 0.12^n.s.^ | 0.27^n.s.^ | 0.54^**^ | 0.44^**^ | 0.53^**^ |
| NBI | 0.64^**^ | 0.62^**^ | 0.57^**^ | 0.62^**^ | 0.67^**^ | 0.68^**^ | 0.14^n.s.^ | 0.16^n.s.^ | 0.18^n.s.^ |
| ExR | -0.20^n.s.^ | 0.17^n.s.^ | -0.34^*^ | -0.51^**^ | -0.63^**^ | 0.28^n.s.^ | **-0.72^**^** | -0.70^**^ | **0.78^**^** |
| ExG | -0.62^**^ | -0.58^**^ | -0.42^**^ | -0.46^**^ | -0.46^**^ | -0.48^**^ | 0.07^n.s.^ | 0.03^n.s.^ | 0.03^n.s.^ |
| G/R | 0.01^n.s.^ | 0.13^n.s.^ | 0.26^n.s.^ | 0.41^**^ | 0.61^**^ | 0.39^**^ | 0.67^**^ | 0.59^**^ | 0.72^**^ |
| G/B | -0.57^**^ | -0.59^**^ | -0.43^**^ | -0.48^**^ | -0.52^**^ | -0.56^**^ | 0.27^n.s.^ | 0.33^*^ | 0.15^n.s.^ |
| R/B | -0.66^**^ | -0.66^**^ | -0.58^**^ | -0.56^**^ | -0.68^**^ | -0.75^**^ | 0.12^n.s.^ | 0.21^n.s.^ | -0.29^*^ |
| GMR | 0.16^n.s.^ | 0.13^n.s.^ | 0.40^**^ | 0.26^n.s.^ | 0.41^**^ | 0.32^*^ | 0.64^**^ | 0.51^**^ | 0.68^**^ |
| INT | -0.35^*^ | 0.19^n.s.^ | 0.03^n.s.^ | 0.24^n.s.^ | -0.54^**^ | 0.02^n.s.^ | -0.59^**^ | **-0.81^**^** | 0.54^**^ |

**Note:** n.s., ^*^, and ^**^ represent ‘not significant’, p<0.05, and p<0.01, respectively.

**Supplementary Table 4**

The range of statistical indicators of *R^2^*, *RMSE* and *NRMSE* obtained from different flight altitudes and GMR segment thresholds across growth stages.

| Flight altitudes | Statistical indicators | Booting stage | | |  | Flowering stage | | |  | Filling stage | | |  | All | | |
| --- | --- | --- | --- | --- | --- | --- | --- | --- | --- | --- | --- | --- | --- | --- | --- | --- |
|  |  | Min | Max | CV |  | Min | Max | CV |  | Min | Max | CV |  | Min | Max | CV |
| 50m | R^2^ | 0.63 | 0.66 | 1.2% |  | 0.59 | 0.62 | 1.2% |  | 0.60 | 0.61 | 0.83% |  | 0.62 | 0.64 | 0.9% |
|  | RMSE | 0.33% | 0.34% | 1.4% |  | 0.29% | 0.30% | 1.2% |  | 0.30% | 0.31% | 1.3% |  | 0.35% | 0.36% | 0.9% |
|  | NRMSE | 12.7% | 13.1% | 1.4% |  | 13.1% | 13.5% | 1.3% |  | 16.4% | 16.7% | 1.2% |  | 15.7% | 16.1% | 0.8% |
| 100m | R^2^ | 0.64 | 0.69 | 3.3% |  | 0.58 | 0.61 | 1.6% |  | 0.59 | 0.64 | 2.9% |  | 0.63 | 0.66 | 2.3% |
|  | RMSE | 0.30% | 0.33% | 4.2% |  | 0.29% | 0.31% | 2.5% |  | 0.28% | 0.30% | 2.3% |  | 0.34% | 0.36% | 1.8% |
|  | NRMSE | 11.6% | 12.8% | 3.9% |  | 12.9% | 13.6% | 2.4% |  | 15.5% | 16.5% | 2.3% |  | 15.4% | 16.1% | 1.9% |
| 150m | R^2^ | 0.65 | 0.69 | 2.8% |  | 0.61 | 0.63 | 1.01% |  | 0.51 | 0.63 | 7.8% |  | 0.61 | 0.64 | 1.4% |
|  | RMSE | 0.31% | 0.33% | 3.1% |  | 0.27% | 0.28% | 0.8% |  | 0.28% | 0.34% | 6.6% |  | 0.36% | 0.37% | 1.1% |
|  | NRMSE | 11.8% | 12.7% | 3.1% |  | 12.3% | 12.6% | 0.81% |  | 15.7% | 18.9% | 8.1% |  | 16.0% | 16.4% | 1.1% |

**Supplementary Table 5**

Cross-site model performance metrics for the combined growth stage after GMR segmentation.

| Spatial scale | Flight altitude (m) | Train R^2^ | Test R^2^ | Train RMSE | Test RMSE | Train NRMSE | Test NRMSE |
| --- | --- | --- | --- | --- | --- | --- | --- |
| Leaf | - | 0.72 | 0.59 | 0.32% | 0.37% | 12.3% | 16.8% |
| Canopy | - | 0.63 | 0.45 | 0.34% | 0.48% | 15.1% | 19.8% |
| Plot | 50 | 0.48 | 0.40 | 0.43% | 0.47% | 16.7% | 21.4% |
|  | 100 | 0.52 | 0.39 | 0.41% | 0.46% | 16.1% | 21.1% |
|  | 150 | 0.52 | 0.38 | 0.42% | 0.46% | 16.2% | 20.9% |
